# Novel Sandwich-Structured Hollow Fiber Membrane for High-Efficiency Membrane Distillation and Scale-Up for Pilot Validation

**DOI:** 10.3390/membranes12040423

**Published:** 2022-04-14

**Authors:** Marn Soon Qua, Yan Zhao, Junyou Zhang, Sebastian Hernandez, Aung Thet Paing, Karikalan Mottaiyan, Jian Zuo, Adil Dhalla, Tai-Shung Chung, Chakravarthy Gudipati

**Affiliations:** 1 Separation Technologies Applied Research and Translation Centre (START), Nanyang Technological University–NTUitive Pte Ltd., Nanyang Technological University, Singapore 637141, Singapore; ananda.qua@ntu.edu.sg (M.S.Q.); zhao_yan@ntu.edu.sg (Y.Z.); junyou.zhang@century-water.com (J.Z.); sebastian.hs@ntu.edu.sg (S.H.); aungthetpaing@ntu.edu.sg (A.T.P.); karikalan@ntu.edu.sg (K.M.); adil.dhalla@ntu.edu.sg (A.D.); 2 Food, Chemical and Biotechnology Singapore Institute of Technology, Singapore 637141, Singapore; zuolezl@gmail.com; 3 Department of Chemical and Biomolecular Engineering, National University of Singapore, Singapore 637141, Singapore; 4 Graduate Institute of Applied Science and Technology, National Taiwan University of Science and Technology, Taipei 10607, Taiwan

**Keywords:** PVDF, hollow fiber membranes, vacuum membrane distillation, flux, liquid entry pressure, wastewater treatment, desalination

## Abstract

Hollow fiber membranes were produced from a commercial polyvinylidene fluoride (PVDF) polymer, Kynar HSV 900, with a unique sandwich structure consisting of two sponge-like layers connected to the outer and inner skin layers while the middle layer comprises macrovoids. The sponge-like layer allows the membrane to have good mechanical strength even at low skin thickness and favors water vapor transportation during vacuum membrane distillation (VMD). The middle layer with macrovoids helps to significantly reduce the trans-membrane resistance during water vapor transportation from the feed side to the permeate side. Together, these novel structural characteristics are expected to render the PVDF hollow fiber membranes more efficient in terms of vapor flux as well as mechanical integrity. Using the chemistry and process conditions adopted from previous work, we were able to scale up the membrane fabrication from a laboratory scale of 1.5 kg to a manufacturing scale of 50 kg with consistent membrane performance. The produced PVDF membrane, with a liquid entry pressure (LEPw) of >3 bar and a pure water flux of >30 L/m^2^·hr (LMH) under VMD conditions at 70–80 °C, is perfectly suitable for next-generation high-efficiency membranes for desalination and industrial wastewater applications. The technology translation efforts, including membrane and module scale-up as well as the preliminary pilot-scale validation study, are discussed in detail in this paper.

## 1. Introduction

Freshwater scarcity is becoming a major challenge for meeting requirements toward basic human needs for agriculture and industry, impeding efforts to meet the global water demand due to an increase in population and industrialization, particularly in coastal countries due to the lack of sufficient fresh water sources or storage capacity [1,2,3,4,5]. Only 3% of the water on Earth is considered fresh water and only 1.2% has potential use as drinking water because the rest is locked in glaciers, ice caps, and permafrost. To deal with water scarcity and freshwater shortage, seawater desalination processes are being widely used. The current solutions utilized for producing drinking water from seawater largely rely on thermal methods such as multistage flash and multi-effect distillation (MSF and MED, respectively) [5,6,7,8] as well as pressure-driven seawater reverse osmosis (SWRO) [8,9], which comprises about 70% of the desalination processes worldwide. However, both thermal and pressure-driven RO technologies are extremely intensive in terms of energy consumption and, currently, they strongly depend on fossil fuels for their operation [8,9]. In addition, although the brine resulting from the SWRO process still has the potential for additional water recovery, it is very difficult to treat [10,11].

Another potential freshwater source is found through wastewater reclamation. In this process, the use of reverse osmosis (RO) by itself is not enough and using conventional processes or a combination of these with RO systems tends to be costly and the purification ineffective [11,12,13]. Due to these reasons, alternative desalination technologies and the use of renewable energies are being researched and developed to reduce the energy consumption and improve overall process productivity [14,15,16,17,18]. 

Membrane distillation (MD) is a promising technology that can potentially compete with the existing MSF/MED and SWRO solutions and could be capable of overcoming the issues previously described [17,19,20]. The key benefits of using MD technology include: (1) less stringent operation conditions compared to conventional desalination, including lower vacuum, or pumping pressures, (2) higher rejection of salts (theoretically approaching 100%), (3) larger contact areas in smaller modular footprints, and (4) ability to treat extremely high salinities of feed water beyond the SWRO tolerance limit. Salts and non-volatile compounds are rejected by the membrane and could produce an even more concentrated brine than the one typically obtained by the RO processes, facilitating efforts toward zero liquid discharge [21,22,23]. This latter characteristic is an advantage because, in theory, one can have higher recoveries using MD compared to RO. Finally, (5) the use of low-grade heat sources, which opens the possibility of the use of renewable energy sources [10,18,20,24,25].

In membrane distillation, the membrane is one of the key factors that govern the separation process in different applications. The MD membrane works as a barrier separating a feed water solution from the permeate vapor stream and simultaneously transfers the vapor produced from the feed stream through the membrane wall to the permeate stream. The permeate stream is condensed on the permeate side or externally using different techniques. MD is a process based on the change in phase due to a thermal gradient allowing the separation of the volatiles, in this case, water from the feed solution, and applying the principles of vapor–liquid equilibrium and heat and mass transfer [24,26]. An ideal MD membrane should be highly porous and hydrophobic, with a very tight pore size distribution and small pore size, and it should have cheap and easy fabrication conditions for large-scale production. Many technology breakthroughs using hydrophobic materials in both flat sheet and hollow fiber membrane configurations have been accomplished, including polyvinylidene fluoride (PVDF), polytetrafluoro-ethylene (PTFE), and polypropylene (PP) [27,28]. 

The techniques using MD to recover water vary according to what is used in the permeate side to drive the separation; these include Direct Contact Membrane Distillation (DCMD), where the permeate side is in contact with cold pure water coming from the DCMD process itself. In Air Gap Membrane Distillation (AGMD) and Sweeping Gas Membrane Distillation (SGMD), air or an inert gas is used to collect water vapor that will be condensed in situ or externally, respectively. In vacuum membrane distillation (VMD), water vapor is removed by applying vacuum on the permeate side and is subsequently condensed externally, similar to the SGMD process [12,17,21]. Other liquids and materials have been used in MD configurations, which are combinations of the above techniques [25]. 

From these configurations, VMD is one of the most attractive MD processes for water reclamation purposes due to its lower costs in operation. These costs are related to fewer stringent mechanical properties needed for the membrane material given that VMD needs lower temperatures and pressures to work with, including pumping and vacuum pressures [29,30]. Using vacuum boosts the water flux due to the gradient in vapor pressure across the membrane and improves heat and mass transfer as there are no separation media involved compared to DCMD, for example [31,32]. These characteristics make VMD suitable for decreasing energy consumption by replacing SWRO or complementing it using the SWRO brines to extract even more freshwater [29].

The main shortcomings of using vacuum are that the vacuum pressure cannot exceed the liquid entry pressure (LEP_w_) to avoid membrane wetting and breaking. Another issue is the energy consumption related to the heating of the feed solution and the condensation of the permeate [24,32]. To assess these drawbacks, several works using hollow fiber membranes have addressed different methods to increase the efficiency of the membranes. The most relevant is membrane fabrication through a spinning process that includes the formulation of the dope composition and the spinning process itself, conducted by thermally induced phase separation (TIPS) or non-solvent induced phase separation (NIPS) [25]. 

The dope composition coupled with the preparation are the main obstacles that need to be overcome for the transition from a bench-scale to a commercial product. PVDF has been employed for MD membrane manufacturing via NIPS due to its processing versatility, hydrophobicity, and resistance to a wide range of chemical products [25]. However, the LEP_w_ values and the mechanical strength of such PVDF membranes have proven to be unsuitable for scaling up as commercial products. A good hollow fiber PVDF membrane must have a spongy internal structure with a reduced presence of macrovoids, which are important to avoid membrane malfunction when they are working under high stresses from the feed pressure and the vacuum. However, there must be a balance, as a more open structure (low tortuosity, higher porosity including macrovoids, and pore size) increases flux, whereas the opposite is important to prevent membrane wetting and failure [33,34]. 

To design a PVDF hollow fiber membrane for MD with greater mechanical strength and excellent hydrophobic properties, it is necessary to tackle some intrinsic characteristics of the dope, specifically the viscosity, which affects the phase inversion and the general structure of the membranes [25,35]. The concentration of the polymer as well as the solvents and additives must be formulated to have the correct balance as previously described, and this has to be coupled with an optimized set of phase inversion conditions [33,34,35,36,37]. 

The present article is primarily focused on scaling up a patented MD technology for manufacturing PVDF hollow membranes, which have a unique sandwich structure, from laboratory scale to commercial scale and producing commercial size modules for pilot validation [19,20,28,37]. Furthermore, the effects in the final product characteristics of the PVDF source in the dope formulation, the coagulation bath composition and temperature, the bore fluid temperature, and the high-speed spinning are investigated in this work. After the membranes were successfully manufactured, they were used for module fabrication at various scales, namely 0.5-inch modules (bench scale) and 2-inch modules (large scale). Two-inch modules were further tested for ≥100 h in a VMD pilot plant, simulating a seawater desalination process using NaCl solutions (≈35 g/L).

## 2. Materials and Methods

### 2.1. Materials

All chemicals used during the membrane fabrication and scale-up were of industrial and reagent grade and used without further purification. Polyvinylidene fluoride (PVDF 1, PVDF 2) (Kynar HSV 900 PWD resin, Arkema, Calvert City, KY, USA and Changshu, China, respectively); lithium chloride (LiCl) (GCE Laboratory Chemicals–TACT Chemie S.E.A. Pte. Ltd., Singapore); N-Methyl-2-Pyrrolidone (NMP) (Puyang Guangming Chemicals Co., Ltd., Puyang city, China); ethylene glycol (EG) (TACT Chemie S.E.A. Pte. Ltd., Singapore); methanol (MegaChem Ltd., Singapore); hexane HPLC grade (Fisher Scientific, Fairlawn, NJ, USA); sodium chloride (NaCl) (Pure Dried Vacuum Salt, INEOS Enterprises, Runcorn, UK). Deionized water was acquired from a PURELAB Option-Q DV 25 unit from ELGA with a resistivity of 18.2 MΩ·cm.

### 2.2. Polymer Characterization

The molecular weights of the two PVDF 1 and PVDF 2 polymers were not provided by the manufacturer. However, the gel permeation chromatography (GPC) of the commercial Kynar^®^ HSV900 has been reported to contain two peaks corresponding to the number-average molecular weights (M_n_); one at ~92,840 kDa (24.92%) and another one at ~1367 kDa (75.08%) [38]. It is possible that the molecular weights of the two polymers may differ from each other due to process variations at different locations. The potential differences in the molecular weights of PVDF 1 and PVDF 2 are reflected in the slightly different solution viscosities measured at the spinning temperature (~50 °C), as shown in Table 1.

These samples were analyzed using differential scanning calorimetry (DSC), thermogravimetric analysis (TGA), and pyrolysis–gas chromatography–mass spectrometry (pyrolysis-GCMS). 

The DSC analysis (Q20, TA Instruments, New Castle, DE, USA) was performed in a dry nitrogen atmosphere. Around 5–10 mg of powder was tightly encapsulated into an aluminum pan. The melting behavior of polymer/diluent samples was analyzed after equilibrating the sample at 40 °C and then heating it at a rate of 10 °C/min until reaching a temperature of 250 °C, and subsequently sustaining this value for 2 min. The crystallization curve was later obtained by cooling the sample to 40 °C at a rate of 10 °C/min after equilibrating at 250 °C for 2 min. The thermogravimetric analysis (TGA) was conducted using a thermal analyzer (SDT Q600, TA Instruments, New Castle, DE, USA) under a nitrogen flow at 100 mL/min. The samples were tested after equilibrating the sample at 40 °C and then heating it in a temperature range of 40–700 °C at a rate of 20 °C/min with an isothermal treatment at the end point for 5 min.

To perform the pyrolysis-GCMS tests, a GCMS-Pyrolyzer (Agilent Technologies 7890B GC, Agilent Technologies 5977A MSD, Frontier Lab Multi-Shot Pyrolyzer EGA/PY3030D) was used. The analysis cup containing a 0.2 mg sample was inserted into the Multi-Shot Pyrolyzer EGA/PY3030D. Samples were pyrolyzed at 600 °C for 1 min. Pyrolysis products were injected with a split of 50 using the Agilent Technologies 7890B GC (equipped with an Ultra ALLOY-5 column (30 m, 0.25 mm, 0.25 mm film of 5% diphenyl–95% dimethylpolysiloxane) (Frontier Lab). The temperatures of the pyrolizer interface and the injection port were both set at 300 °C. Helium was used as a carrier gas with a constant flow of 1 mL/min. The initial oven program was set as follows: 40 °C for 2 min, then increased to 320 °C at 20 °C/min and then maintained for 13 min. Mass spectra were obtained by the Agilent Technologies 5977A MSD. The interface temperature was set at 300 °C, the ion source temperature was set at 230 °C, the ionization voltage was set at 70 eV, and a mass range from 33 to 600 m/z was scanned at a scan speed of 1526 μ/s.

### 2.3. Fabrication of Hollow Fiber Membranes

The PVDF hollow fiber membranes were fabricated with a formulation of the polymer dope and spinning conditions developed by Zuo and Chung [28,37]. The spinneret used is a dual-layer spinneret with a bore output of 0.44 mm and an inner channel between 0.6 and 1.14 mm. The bore fluid was fed from the top and the dope from the side of the spinneret. Table 2 summarizes the spinning parameters, such as line speed, air-gap distance, dope flowrate, and bore fluid flowrate, and includes temperatures of the dope, bore liquid solution, and the coagulation bath. However, some of the conditions for the mix of the dope and the spinning of the membranes were modified to adapt the process to large-scale production and have consistent results. Briefly, PVDF 1 and PVDF 2 were mixed separately in each batch for 24 h at 65 °C. Then, (1) each dope was degassed for another 48 h in the reactor to guarantee complete dissolution of the polymer and removal of entrapped air bubbles in the mix; (2) the take-up speed (line speed) of the fiber and the temperature of the coagulation bath were optimized during the spinning process; (3) dope and bore flowrates were adjusted to the line speed and to obtain similar results as the baseline work cited; (4) after spinning, the new fibers were stored in water for 3 days to remove the residual solvents; (5) the membranes were post-treated with alternate baths of methanol followed by hexane to remove the water from the fibers and increase hydrophobicity; (6) the membranes were dried in a dry room at room temperature (RT) at least two days before being inspected and selected for testing and module production. 

As part of the initial scale-up trials from a lab-scale fabrication line to pilot-scale production, two different PVDF (PVDF1 and PVDF 2) sources were identified based on the prior data, cost of materials, and ease of availability for large-scale production. Different spinning conditions were employed to optimize the membrane fabrication process, which could be scaled-up from small 1.5 kg batch sizes to 50 kg batch sizes. The coagulation bath temperatures and the bore fluid flowrates were varied for both the PVDF materials employed, as shown in Table 3. 

The temperatures of the polymer dope were constantly monitored during mixing, degassing, and spinning. The spinning required up to three working days for batch sizes of ≥20 kg of dope, which required degassing at the end of each working day. The viscosity of the different dopes was measured close to the spinning temperature of 55 °C using a viscometer (Cole-Palmer VCPL 340015, Vernon Hills, IL, USA).

The membranes were characterized with a Field Emission Scanning Electron Microscope (FESEM) (JEOL JSM-7200F) operated at 5.0 kV of accelerating voltage. A goniometer (OCA15EC, DataPhysics Instruments, Filderstadt, Germany) was used to test the static water contact angle of each membrane using the sessile drop method. A droplet of deionized water was mechanically pipetted onto the membrane surface and a static image of the droplet on the membrane surface after the equilibrium was taken. This was repeated five times at different locations of the membrane and the average results were reported. The optical images of hollow fibers were obtained using a Leica DVM6 optical microscope.

The pore size distribution was determined by a capillary flow porometer (CFP 1500AEX, Porous Material. Inc., Ithaca, NY, USA), whose working principle was based on the bubble-point and gas permeation tests. The hollow fiber samples were potted into the sample holder and soaked by the wetting fluid (Galwick, with surface tension 15.9 × 10^–3^ N/m) until completely wet. During the test, the gas flowrate was increased stepwise and passed through the saturated sample until the applied pressure exceeded the capillary attraction of the fluid in the pores. By comparing the gas flowrates of both wet and dry samples at the same pressures, the percentage of flow passing through the pores larger than or equal to the specified size can be calculated from the pressure–size relationship. The mechanical properties of hollow fiber membranes were examined using a universal tensile tester (Instron 3342, Norwood, MA, USA). Each specimen was firmly clamped by the testing holder and pulled longitudinally at an elongation rate of 50 mm/min at room temperature. The corresponding mechanical properties were determined by the built-in software.

In another method, the contact angle was determined using a tensiometer (DCAT11 Dataphysics, Filderstadt, Germany). The contact angle quantifies the wettability of a solid surface by a liquid. The sample was inserted into an electro balance for cyclical immersion into DI water. The contact angle was calculated from the wetting force using Wihelmy’s method. The overall porosity of membranes was determined by the gravimetric method with the following Equation (1):

(1) $$\begin{array}{cc} Porosity=1- & \frac{Volume_{Polymer}}{Volume_{total}}\\ & =1-\frac{Membrane\;weight/Membrane\;volume\;}{Polymer\;density} \end{array}$$

where the PVDF density was 1.78 g/cm^3^ and the membrane volume was calculated based on OD and ID of the fibers.

LEP_w_ was determined using dead-end hollow fiber modules containing a single membrane fiber. LEP_w_ measures the pressure required to force water through the pores of a dried membrane and is an indication of how easily a hydrophobic membrane could be wetted. Water was gradually pressurized at a 0.5 bar increment. As water pressure was increased, water could be pushed out of the membrane pores, and the pressure at which water droplets were visible on the outer surface of hollow fibers was recorded as the LEP_w_ of the membranes.

### 2.4. Membrane Module Testing

**Figure 1:**
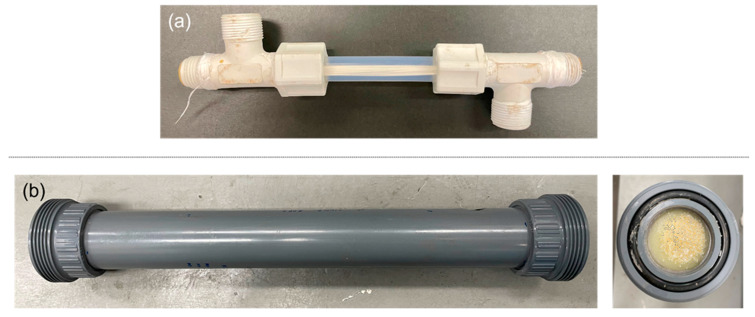
Scale-up of membrane distillation modules. (**a**) Lab-scale testing module (0.5-inch diameter); (**b**) pilot-scale testing module (2-inch diameter).

**Figure 2:**
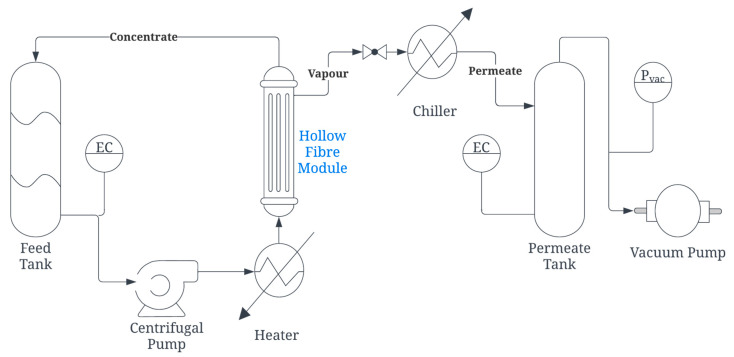
Process flow schematic for the vacuum membrane distillation (VMD) used for testing 2-inch modules at START.

The hollow fibers provided were assembled into 0.5-inch diameter or 2-inch diameter modules, as shown in Figure 1, and tested at the Environment & Water Innovation Centre of Innovation (EWTCOI) and our facility, Separation Technologies Applied Research and Translation Centre (START), respectively. For the 0.5-inch modules, after the target temperature of the feed was reached, temperature sensors for the feed inlets and outlets were calibrated. The feed water was recirculated through the lumen side of the hollow fibers. The liquid feed entered the module in an upward direction to minimize air bubbles in the module. Once the feed inlet temperature in the membrane module reached a steady state, the vacuum pump was switched on to create a vacuum in the shell side of the hollow fibers. The timer for permeate collection was started and permeate was collected by condensing the water vapor either in an ice chip bath, which was periodically refilled with ice chips (0.5-inch modules) or using a chiller at 15 °C (2-inch modules). The amount of permeate collected was gravimetrically determined using a weighing scale and the electrical conductivity (EC) was measured. Table 4 shows the conditions for each of the tests and Figure 2 presents a process flow schematic for the in-to-out setup of VMD used in this study, which was a semi-continuous operation with variation in EC. The feedwater was filled with a NaCl solution whenever the EC value was nearly doubled or the tank was at half capacity, whichever came first. Each time the feed was filled, the vacuum was switched off until the target temperature of the feed was reached again.

## 3. Results and Discussion

### 3.1. Characterization of Polymers and Dopes

The two samples of PVDF1 and PVDF 2 were similar according to their melting, crystallization, thermal degradation temperatures, and pyrolysis–GCMS chromatograms, as shown in Table 1 and in Figure S1 in the Supplementary Materials, respectively. Melting transition temperatures differed from those reported for pure PVDF (177–179 °C) but were close to the reported values for commercial samples, from 159 °C to 173 °C [39,40,41]. Rapid crystallization was demonstrated by sharp and narrow peaks with a degree of supercooling of about 35 °C difference from the melting points for both samples, as seen in Figure S1. These crystallization points were lower than the ones reported for pure PVDF, which are between 139 °C and 141 °C, at the same rate of cooling [42].

Based on the TGA results, differences with pure PVDF were also observed in its maximum temperature for thermal degradation [43,44]. However, there were no differences among the samples examined, as shown in Figure S2 for the TGA thermograms. In the case of the pyrolysis–GCMS, the peaks on the chromatograms differed by the number of counts, but the times of separation were the same. The repeating unit of PVDF, vinylidene fluoride, was separated after 2.5 min for both samples PVDF 1 and PVDF 2. On the other hand, melting enthalpy, crystallization enthalpy, and the viscosity of the dope showed a clear difference between the two PVDF samples, as well as with the reported value of pure PVDF (104.7 J/g) [45]. These differences have an impact on the performance of the final product characteristics and could be correlated to differences in molecular weight, polydispersity, or the branching of the PVDF chains [39].

### 3.2. Characterization of PVDF Hollow Fiber Membranes

The bore fluid flowrate and coagulation temperature were the initial factors that were chosen to conduct an experimental design based on the previous work [28,37]. The first and second batches were conducted using a polymer dope of 1.5 kg. The dope B1 and B2 were made using PVDF 1 and PVDF 2, respectively. For the batch B1, the dope flowrate was kept constant at 4.5 mL/min and the take-up line speed at 3.0 m/min. The process conditions for the 1.5 kg batch sizes were optimized by varying the coagulation bath temperature and the bore fluid flowrate, as shown in Table 3.

**Figure 3:**
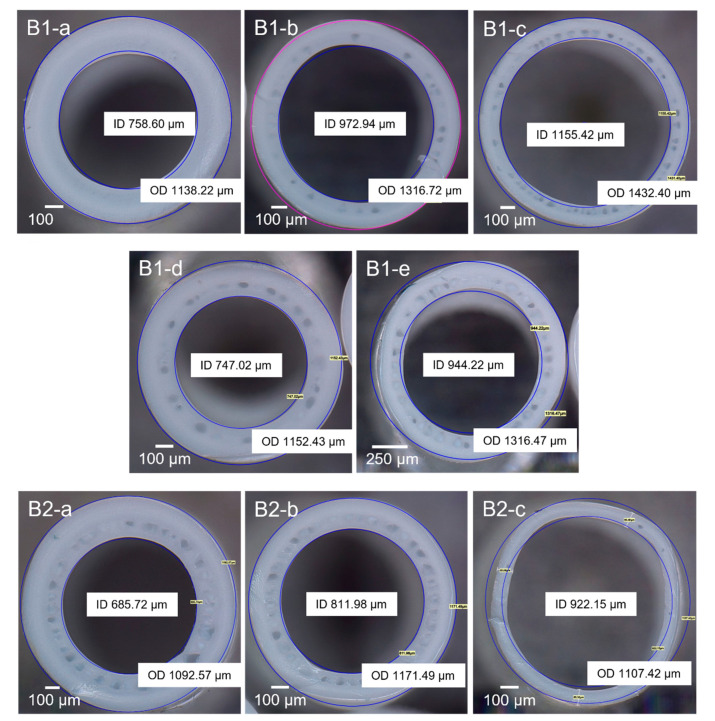
Changes in membrane morphology with changes in spinning conditions. B1: samples from PVDF 1 dope. B2: samples from PVDF 2 dope.

The membranes fabricated under these conditions were visually examined using an optical microscope, and the fiber images are displayed in Figure 3 (B1-a to B1-e). The membranes were visually examined and their performance in handling during spinning was also checked. From B1-a to B1-c, the coagulation bath was kept at room temperature (≈24 °C). Membrane B1-a showed the highest strength (i.e., did not break nor collapse during spinning) with the highest thickness and the smallest dimensions (i.e., internal diameter, ID, and outer diameter, OD). Membrane B1-b was in the middle of B1-a and B1-c in terms of dimensions and showed the characteristic sandwich structure, with a thin layer of small-size macrovoids that was present in all subsequent batches. B1-c possessed a softer structure than B1-a as it easily collapsed due to handling during spinning; therefore, B1-c spinning conditions were not retested. Membranes B1-d and B1-e had the coagulation bath at a higher temperature. B1-d had a similar dimension to B1-a, while the structure changed to a more porous one than B1-a due to the higher coagulation bath temperature. The results are consistent with numerous similar observations reported in the literature [46,47,48]. It has been well established that an increase in coagulation bath temperature results in a faster solvent–non-solvent exchange. Consequently, it leads to a more porous structure, while a slower de-mixing at lower temperatures results in a denser film [49]. Membranes B1-d and B1-e were fabricated at a higher coagulation bath temperature of ~40 °C and bore fluid flowrates of 1.5 and 3.0 mL/min, respectively. As shown in Table 3, the increase in bore fluid flowrate resulted in an increase in ID and OD of the membrane B1-e. A similar trend was also observed for membranes B1-a, B1b and B1-c, where the diameter increased as the bore fluid rate increased from 1.5 to 4.5 mL/min, also leading to a reduced wall thickness. Membrane B1-e started to lose its round shape and the membrane strength significantly decreased when elevating the bath temperature. From these results, it is revealed that the membrane dimensions increase and thicknesses reduce when the bore flowrate is boosted [25,50].

As part of our efforts to scale-up the membrane fabrication process from a lab scale to a pilot scale, the batches with PVDF 2 were run at a higher take-up speed of 9 m/min and, consequently, the dope flowrate had to be adjusted to 13.5 mL/min to be consistent with the lower line speeds used for PVDF 1 batches. As shown in Table 3, membrane B2-a had the highest strength and the highest thickness of the batch. Membrane B2-b had similar features as B2-a but with a slightly higher ID. Membrane B2-c appeared deformed due to its small membrane wall thickness and, therefore, these spinning conditions were not considered for further experiments. Like the previous batch results, when the bore flowrate was boosted, the membrane dimensions increased and the thicknesses reduced. As evident from Table 3, the increased bore fluid flowrate resulted in larger diameters (ID and OD) as well as a lower wall thickness, eventually leading to the loss of mechanical integrity (for B1-c and B2-c). As the bore fluid flowrate increased, the solvent–non-solvent exchange rate increased, leading to higher mass transfer and faster polymer de-mixing. The higher bore fluid flowrate also radially expanded the fiber dimensions and thinned the fiber wall, thus reducing the overall mechanical strength [50,51,52].

The microscopy images and the very feasible optimization of the membranes suggested that membranes B2-a and B2-b had the potential to be scaled-up to 20 kg and subsequently 50 kg batches. Based on the performance results, it was determined that the conditions used for the membrane B2-a were most suitable for the final scale-up stage when using PVDF 1 in the polymer dope instead of PVDF 2.

**Figure 4:**
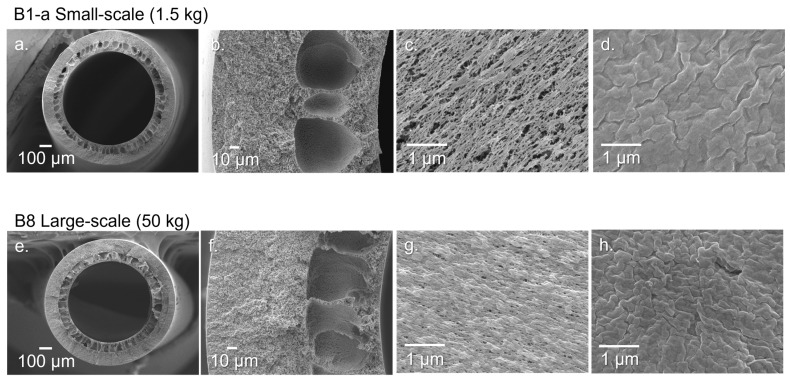
FESEM of PVDF hollow fiber membranes. (**a**,**e**) Cross-section of membrane; (**b**,**f**) zoom-in of membrane’s cross-section; (**c**,**g**) inner surface of membrane; (**d**,**h**) outer surface of membrane.

Figure 4 shows the FESEM images of the membrane samples from a small batch size (1.5 kg, B1-a) and a large production-scale batch size (50 kg, B8). The SEM images confirm the formation of the novel sandwich-like structure with a porous inner layer filled with macrovoids between two thin, denser outer layers. They are consistent with the previously reported literature [19,33,37].

The sandwich structure, with the two sponge-like layers, improves the mechanical properties, and increases the evaporation area and the vapor transport during the VMD process in an in-to-out configuration. The rapid de-mixing in the outer layer is due to the use of water as a non-solvent, which produces a closer porous structure than the inner layer that helps to avoid membrane wetting due to condensation in the permeate side. The inner surface is more porous due to the use of an NMP/water solution (50/50 wt./wt.) as the bore fluid, which delays the phase inversion. LiCl and EG are used to decrease the miscibility of the solvent in the dope, allowing a more controlled liquid–liquid extraction of the dope. LiCl, by increasing the dope viscosity, also helps to reduce the size of the macrovoids, thereby increasing the strength of the membranes. While the inner surface of B8 is still very porous, it is less porous than the smaller scale batches B2-a and B2-b. This small change in the structure is probably due to the increase in dope viscosity shown in Table 1 when using PVDF 1 as the base polymer.

**Figure 5:**
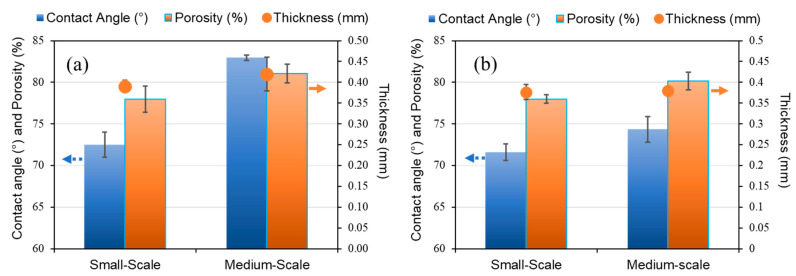
Porosity, contact angle, and thickness of produced hollow fiber membranes in each batch. Small scale: 1.5 kg batch; medium scale: 20 kg batch. (**a**) Fibers made with PVDF 1 dope; (**b**) fibers made with PVDF 2 dope.

The porosity, contact angle, and thickness of the samples increased when the polymer in the dopes was changed from PDVF 2 to PDVF 1, as shown in Figure 5. The membranes made from PVDF 1, prepared using similar spinning and dope conditions as the membrane B-2a (Table 3), showed higher contact angle and porosity values than the ones from PVDF 2 for batch sizes of 1.5 kg and 20 kg, as shown in Figure 5.

**Figure 6:**
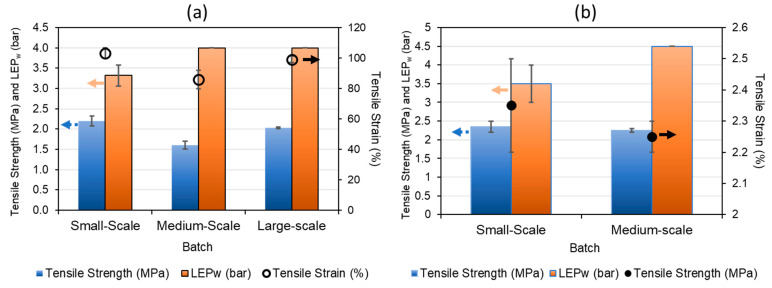
Tensile strength and strain and liquid entry pressure (LEP_w_) of water in produced hollow fiber membranes in each batch. Small scale: 1.5 kg batch; medium scale: 20 kg batch; large scale: 50 kg. (**a**) Fibers made with PVDF 1 dope; (**b**) fibers made with PVDF 2 dope.

On the other hand, the tensile strength decreased when the production was scaled-up, as shown in Figure 6. This behavior could be explained by the constant tension to which the membranes were subjected during the long continuous fabrication process. The spinning process was performed for up to three days due to the larger quantities of dopes, and these conditions could have subtle changes in dope compositions from one day to the next. Here, the tensile strength was proportionally higher when using PVDF 1 in the dope than when PVDF 2 was used, as shown in Figure 6a. The tensile strain also decreased when increasing the batch size using PVDF 1 (Figure 6a). On the contrary, the tensile strain values seemed to increase when using PVDF 2 in the dope. It is worth noting that from batch B3 onwards, the conditions of the spinning process were adopted at larger scales of 20 kg and 50 kg (see Table 2); thus, the values obtained by the large-scale batch (50 kg) show that there was an optimization of conditions that led to an increase in the mechanical properties. In addition, the LEP_w_ values showed more consistency between batches, which is a very important feature for obtaining better results in the VMD process. These findings suggest that large-scale reproducibility is commercially achievable with small changes aiming to increase production.

### 3.3. VMD Tests

Once assembled, the small 0.5-inch modules prepared using PVDF 1 and 2 dopes were placed in a vacuum membrane distillation unit at the EWTCOI facility. These modules were tested by treating a 35 g/L synthetic NaCl feed solution, which was used to simulate seawater to validate the membrane modules for desalination application, as per the operating parameters outlined in Table 4. The modules were prepared using membranes spun using the conditions described in Table 2 and Table 3 in a 1.5 kg batch size, and characteristics such as salt rejection and flux were evaluated in VMD mode (Figure S4). The VMD tests performed on the 0.5-inch modules showed a higher flux for hollow fibers produced from the dope 1 than from the dope 2, with differences of about 20 L/m^2^.h for the same spinning conditions (B3 vs. B5). The salt rejection, based on electrical conductivity (EC) measurements, remained consistent with values close to 100% for all modules tested, as seen in Figure S4. In comparison to the research reported in the literature, the membranes produced in this work showed higher flux values under similar operating conditions [29,32]. It is worth noting that with each batch iteration, the consistency in the membrane characteristics increased and was maintained, especially for the batches that used PVDF 1 (Figure S4a). These VMD results of the 0.5-inch modules from smaller-scale batches confirmed the suitability of PVDF 1 for the full-scale spinning process, as discussed in the previous section.

**Figure 7:**
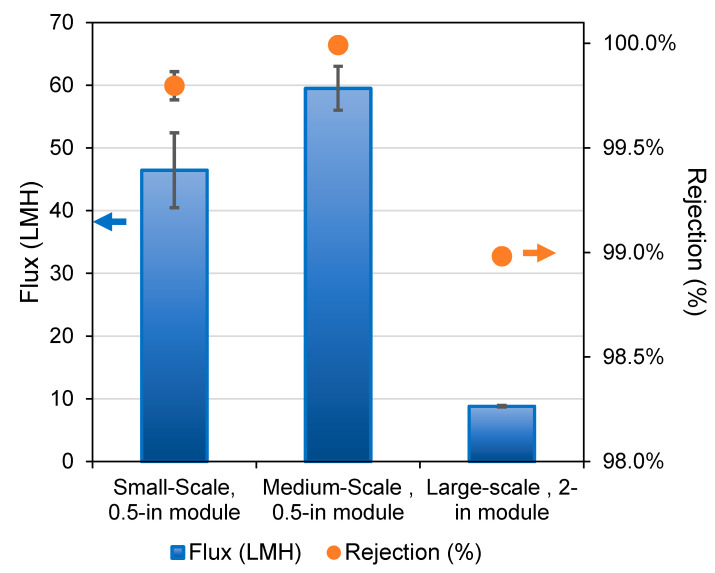
Flux and rejection of VMD tests in each batch using PVDF 1. All tests were performed for time ≥ 1 h using 0.5-inch modules and time ≥ 100 h using 2-inch modules. Small scale: 1.5 kg batch; medium scale: 20 kg batch; large scale: 50 kg.

Once the production conditions were selected, 2-inch modules were assembled (Figure 1b) and tested using a custom-built MD unit capable of operating in VMD and DCMD modes. The process flow diagram for the VMD operation using the skid is shown in Figure 2. Figure 7 depicts the VMD test results for the 0.5-inch and 2-inch modules at EWTCOI and START facilities, respectively. In order to evaluate the reproducibility of the membrane characteristics as a function of dope batch sizes, two sets of 0.5-inch modules were assembled with membranes prepared from small- and medium-size batches (i.e., 1.5 kg and 20 kg dope sizes) and then compared with 2-inch modules assembled with membranes prepared from a large batch size of 50 kg. As shown in Figure 7, the fluxes of small 0.5-inch modules remained high at 47 L/m^2^.h and 60 L/m^2^.hr for membranes prepared from 1.5 kg and 20 kg batch sizes, respectively. However, as the module size is increased to 2-inch, the flux drops significantly to ~10 L/m^2^.h.while the salt rejection remains >90%. The high salt rejection indicates that the membrane’s microporous structure is still intact and reproducible at different batch sizes; the decline in flux may be attributed to module characteristics such as flow pattern, flow distribution, and temperature polarization. This flux decline phenomenon tends to be higher in an in-to-out configuration, thus diminishing the mass and heat transfer efficiencies in 2-inch modules [53,54,55,56].

Due to the limitations of the existing VMD unit, the effects of some operating parameters such as feed flowrate, temperature gradient, pressure differential across the membranes, and temperature polarization coefficient (TPC) were not thoroughly evaluated in the current study. A larger 5000 L/day capacity pilot unit with the requisite engineering design to study the effect of the above-mentioned operating parameters on permeate flux is under construction and the results from VMD testing of 16 4-inch modules will be the subject of a subsequent publication.

**Figure 8:**
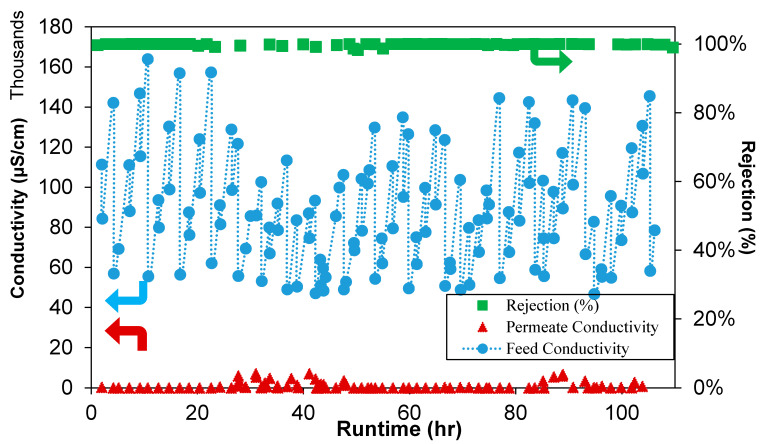
Rejection and conductivity of the feed and permeate as a function of runtime from pilot tests using a 2-inch module.

In order to evaluate the long-term performance and to assess failure modes such as pore wetting under the given test conditions, the 2-inch modules were tested with synthetic seawater prepared with a 35 g/L NaCl, in a batch mode previously described, for over 100 h. The pilot unit was operated for 5–6 h per day with the feed water replenished at the beginning of the day. The flux and the salt rejection data for the 2-inch modules are summarized in Figure 8. Throughout the test duration, the salt rejection and the permeate flux remained consistent at ~100% and within 8–9 L/m^2^·h, respectively, despite the variations in feed concentrations due to the batch mode operations previously described. The stable permeate flux through the test duration and under the given conditions indicates that the membrane pore structure remained intact with no pore wetting, which would have otherwise caused a spike in the permeate conductivity, not seen in this study.

It is important to highlight that the flux of the 2-inch module was nearly six times lower than the highest flux previously reported for the 0.5-inch modules (Figure 7). The significant drop in flux with an increase in module size can be attributed to several factors such as (a) multi-fold increase in the membrane area as well as a much tighter packing density in a larger module, leading to a decrease in the residence time at the feed flowrates employed, (b) a high conductive heat loss leading to the loss of driving force for water vapor transport, and (c) sub-optimal flow distribution either in laminar flow regime or the transition flow regime, all resulting in less efficient mass transfer across the membrane [55,56]. Despite this reduction in flux, the product water flux using the 2-inch module falls within the range of previously reported works where the tests were carried out on a pilot scale [24,54]. These results confirm the long-term effectiveness and high performance of the sandwich-structured hollow fibers developed in this study.

**Figure 9:**
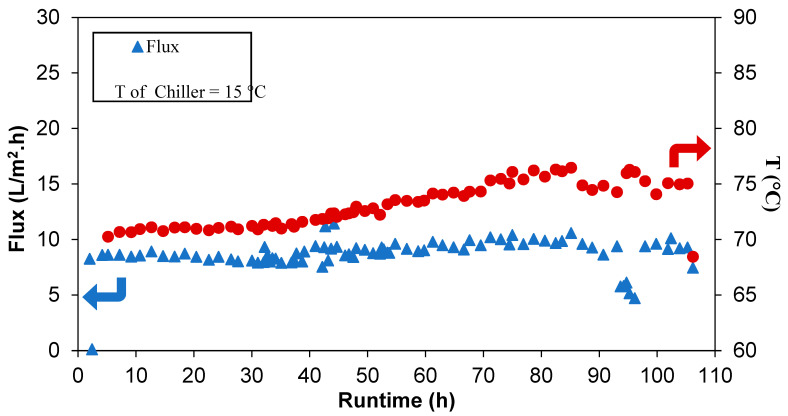
Flux and feed temperature profile from pilot tests using a 2-inch module.

In addition, test conditions such as feed temperatures and flowrates, as well as the test duration, have an impact on the flux (see Table 4 and Figure 9) [55]. For example, the lower feed flowrate used in the 0.5-inch modules increases the residence time, leading to a higher flux. However, these conditions are not suitable for use in industrial or commercial settings because of the very low productivity rates. Nevertheless, the results of the 2-inch modules show fluxes almost four times higher than and comparable rejections to SWRO systems, which are typically in the range of 2.5–3 L/m^2^.h bar and ~99.7% salt rejection, respectively, making these produced hollow fibers suitable alternatives for desalination.

## 4. Conclusions

In this study, we successfully scaled-up a lab-scale membrane fabrication process to produce a novel sandwich-like structure comprising an inner porous layer with controllable macrovoids between two thin layers of sponge-like dense layers. The membrane morphology was optimally designed for membrane distillation applications. The membrane fabrication processes were scaled-up from 1.5 kg batch sizes to 20 kg and 50 kg, clearly demonstrating the feasibility of translating the chemistry and process to a manufacturing set up. The membrane properties such as porosity, mechanical strength, and morphology were optimized by careful control of the spinning conditions. The scaled-up membranes prepared using the optimized conditions were assembled into small 0.5-inch diameter modules with 15 fibers (i.e., an effective membrane area of ~0.0035–0.0051 m^2^) as well as 2-inch diameter modules (i.e., an effective membrane area of 0.46 m^2^), which were tested using MD testing units in VMD mode against a synthetic feed water simulating seawater concentration (35 g/L NaCl). The small modules showed a very high flux of >40 L/m^2^·h under the operating conditions, while the flux drops to ≤10 L/m^2^·h as the module size is increased to 2-inch. Nevertheless, the 2-inch modules tested for over 100 h demonstrated the long-term efficiency of the membranes with a flux maintained at ~8.8 L/m^2^·h while the salt rejection remains close to 100%. These results validate the morphological design employed for the novel PVDF membranes that imparts high mechanical integrity as well as optimal pore structure for highly efficient vapor transport. While the observations are highly encouraging and stand testimony to the suitability of these membranes in applications such as seawater desalination and high-strength industrial wastewater treatment for recycle and reuse, challenges with retaining the flux still linger as the modules are further scaled to a commercial industrial scale of 4-inch or 8-inch diameters. The future efforts of our group are to be extensively focused on module scale-up and field validation using a 5000 L/day pilot unit against actual seawater or industrial wastewater. The results from the pilot validation will be the subject of subsequent publication.

## Figures and Tables

**Table 1:** Characterization of PVDF and dope samples.

Polymer	PVDF 1	PVDF 2
**Melting point (°C)**	162.65	162.64
**Crystallization point (°C)**	127.68	126.81
**Max. thermal degradation (°C)**	472.49	472.66
**Melting enthalpy (J/g)**	36.08	33.92
**Crystallization enthalpy (J/g)**	41.41	36.64
**Dope viscosity (Pa·s)**	101.93 (@50.1 °C)	167.27(@ 51.7 °C)

**Table 2:** Spinning conditions for production of PVDF MD hollow fibers. Based on conditions from Zuo and Chung [28,37]. Design of experiments (DOE) using speed line and coagulation bath temperature as variables. Bore flowrate was adjusted to the DOE parameters.

Batch Number	B1	B2	B3	B4	B5	B6	B7	B8 Onwards
**Dope** **(wt. %)**	PVDF/LiCl/EG/NMP: 13/5/5/77							
**Bore solution** **(wt. %)**	NMP/Water: 50/50							
**Scale-up (kg Dope)**	1.5	1.5	1.5	1.5	20	20	50	50
**Air gap (mm)**	30	30	30	30	30	30	30	30
**PVDF source**	1	2	1	1	2	1	2	1
**Coagulation bath, tap water (°C)**	RT and 40	40	40	40	40	40	40	40
**Line speed (m/min)**	3	9	3	9	9	9	9	9
**Dope flowrate (mL/min)**	4.5	13.5	4.5	13.5	13.5	13.5	13.5	13.5
**Bore flowrate (mL/min)**	1.5–4.5	4.5–9.0	1.5–3.0	4.5–6.8	4.5–6.8	4.5–6.8	4.5–6.8	4.5

**Table 3:** Hollow fiber casting conditions for small 1.5 kg batches using PVDF 1 and PVDF 2.

PVDF	Batch No	Bore Fluid Flowrate (mL/min)	Coagulation Bath Temperature (°C)	Outer Diameter (mm)	Inner Diameter (mm)	Contact Angle (°)
1	B1-a	1.5	≈24	1.13 ± 0.01	0.73 ± 0.01	69.3
1	B1-b	3	≈24	1.29 ± 0.02	0.95 ± 0.01	77.2
1	B1-c	4.5	≈24	1.38 ± 0.01	1.09 ± 0.01	72.3
1	B1-d	1.5	38.3	1.12 ± 0.01	0.70 ± 0.00	66.6
1	B1-e	3	38.3	1.25 ± 0.03	0.89 ± 0.01	64.1
2	B2-a	4.5	38.6	1.07 ± 0.02	0.66 ± 0.03	70.6
2	B2-b	6.8	38.6	1.16 ± 0.01	0.79 ± 0.01	72.6
2	B2-c	9	38.6	1.25 ± 0.01	0.90 ± 0.01	n/a

**Table 4:** Characteristics of testing modules and operating conditions for vacuum membrane distillation (VMD) of water from a NaCl solution. ^†^ Estimated values.

Testing Site	Lab-Scale Module	Pilot-Scale Module
**Module (nominal inches)**	0.5	2
**VMD configuration**	in-to-out	in-to-out
**Number of fibers**	15	560
**Effective length (mm)**	120	370
**Effective membrane area (m^2^)**	0.0035–0.0051	0.456
**Packing density (%)**	≈13 ^†^	35
**Feed flowrate (L/min)**	0.5	8.5–9.5
**Feed temperature (°C)**	88	≥70
**Vacuum (bar)**	−0.80	−0.85
**Test duration (hr)**	≥1	>100
**Feed concentration (g/L NaCl)**	35	≈35.7

## Data Availability

Not applicable.

## Supplementary Materials

The following supporting information can be downloaded at: https://www.mdpi.com/article/10.3390/membranes12040423/s1. Figure S1. DSC of different PVDF powders used for hollow fiber membrane manufacture. (a) PVDF 1, USA origin; (b) PVDF 2, China origin; Figure S2. DSC-TGA of different PVDF powders used for hollow fiber membrane manufacture. (a) PVDF 1, USA origin; (b) PVDF 2, China origin; Figure S3. Pyrolysis–GCMS chromatograms of PVDF at 600 °C. Orange—PVDF 1; green—PVDF 2; Table S1. Characteristics of manufactured hollow fibers. FR: flowrate; CB: coagulation bath; ID: internal diameter; OD: outer diameter; Figure S4. Flux and rejection of VMD tests in each batch. All tests were performed for time ≥ 1 h and using 0.5-inch modules. Descending values based on flux (LMH). (a) Fibers made with PVDF 1 dope; (b) fibers made with PVDF 2 dope.
