# Supplementary material for: Novel Sandwich-Structured Hollow Fiber Membrane for High-Efficiency Membrane Distillation and Scale-Up for Pilot Validation
Source: Membranes (Basel). 2022 Apr 14;12(4):423. doi: 10.3390/membranes12040423 (PMC9032867; doi:10.3390/membranes12040423)
Supplement: Supplementary file 1 [file membranes-12-00423-s001.zip › membranes-1687186-supplementary.pdf]

Appendix A (Supplementrary Data)

Supplementary Materials

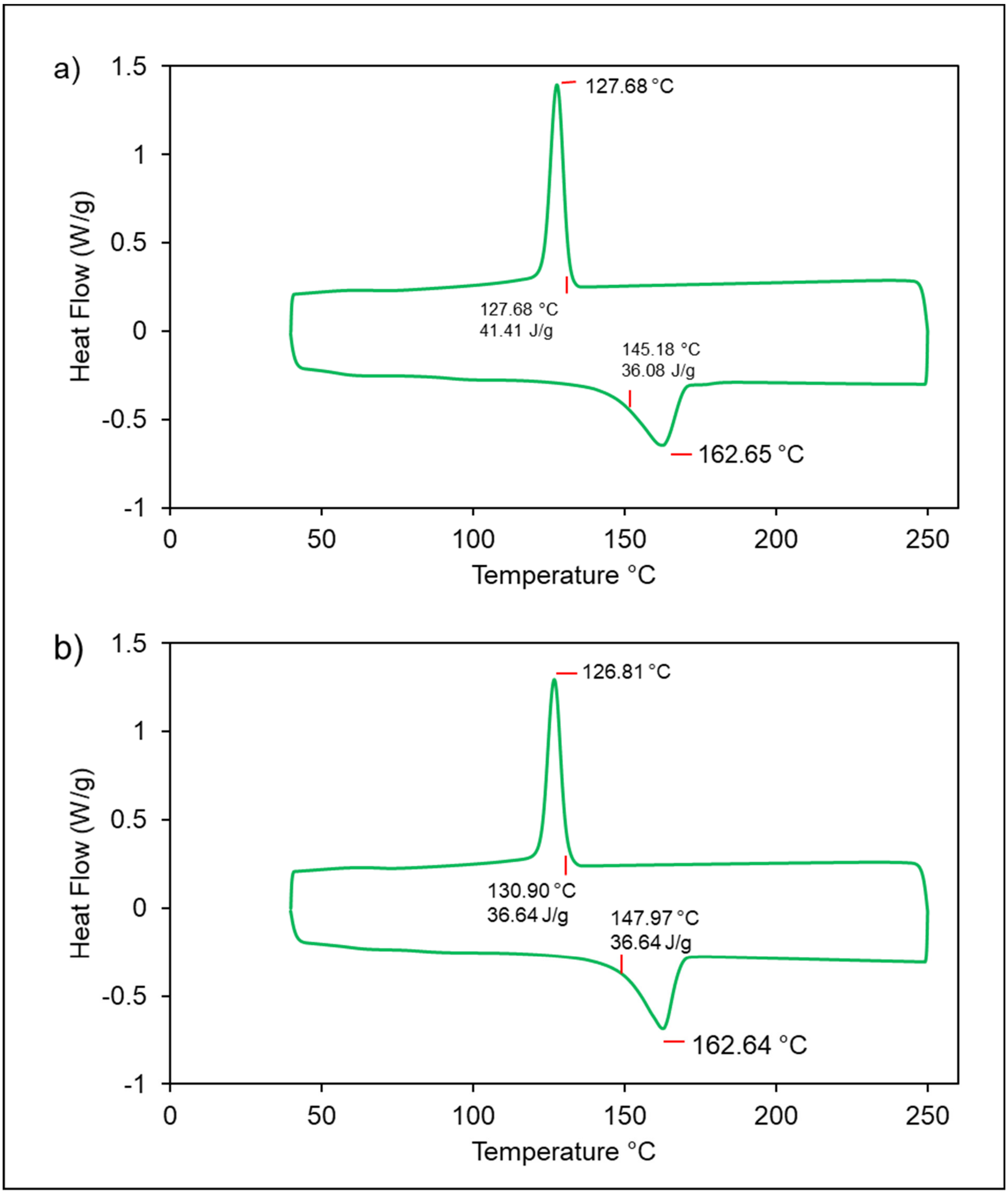

Figure S1. Differential Scanning Calorimetry (DSC) of different PVDF powders used for Hollow fiber membrane manufacture. (a) PVDF 1, USA-origin; (b) PVDF 2, China-origin.

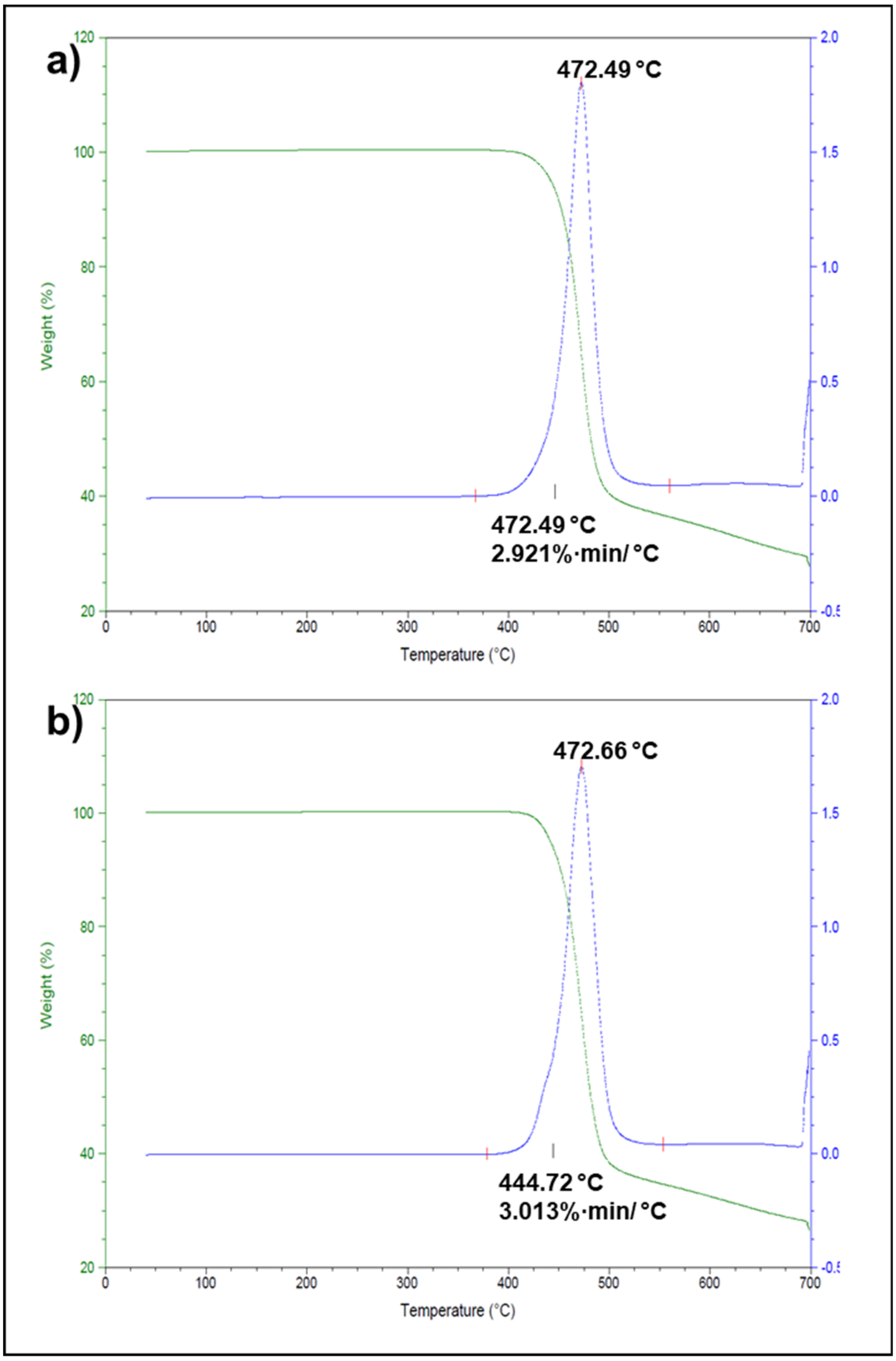

Figure S2. Thermogravimetric analysis (TGA) of different PVDF powders used for Hollow fiber membrane manufacture. (a) PVDF 1, USA-origin; (b) PVDF 2, China-origin.

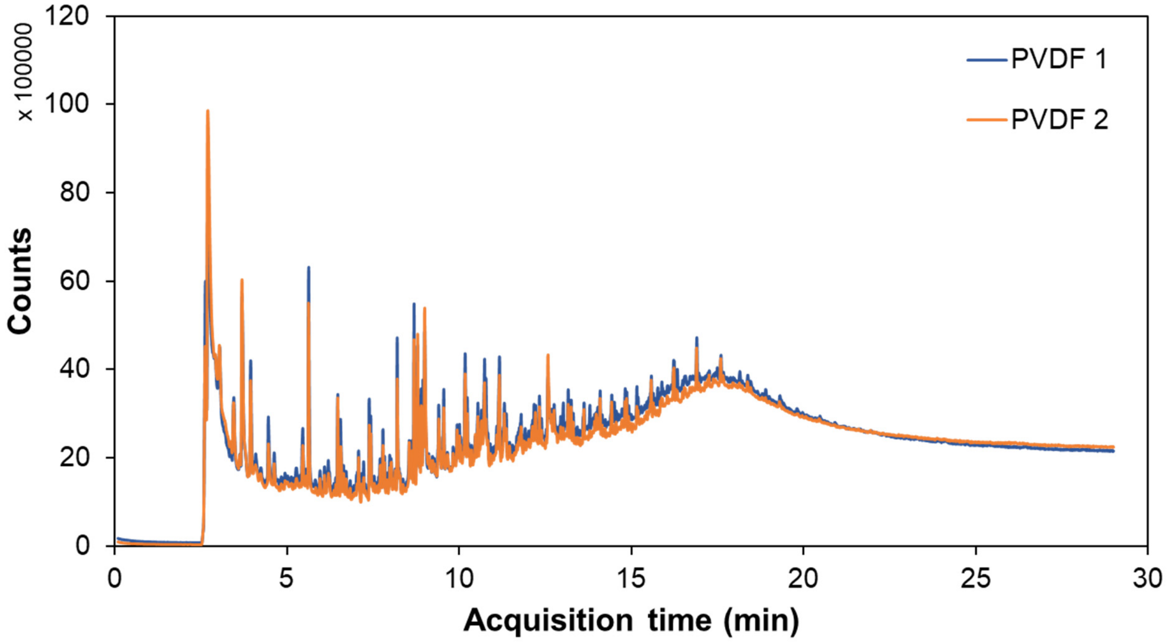

Figure S3. Pyrolysis-GCMS chromatograms of PVDF at 600 °C. Orange-PVDF 1; Green – PVDF 2.

Table S1. Characteristics of manufactured hollow fibers. FR: flowrate; CB: coagulation bath; ID: internal diameter; OD: outer diameter.

Polymer	Batch sample	Bore FR (mL/min)	CB (°C)	OD (mm)	ID (mm)	Porosity (%)	Contact Angle (°)
PVDF 1	**B1-a**	1.5	≈ 24	1.13 ± 0.01	0.73 ± 0.01	n/a	69.3
	**B1-b**	3.0	≈ 24	1.29 ± 0.02	0.95 ± 0.01	n/a	77.2
	**B1-c**	4.5	≈ 24	1.38 ± 0.01	1.09 ± 0.01	n/a	72.3
	**B1-d**	1.5	38.3	1.12 ± 0.01	0.70 ± 0.00	n/a	66.6
	**B1-e**	3.0	38.3	1.25 ± 0.03	0.89 ± 0.01	n/a	64.1
	**B3-a**	3.0	38.3	1.04 ± 0.01	0.63 ± 0.01	75.55 ± 0.2	72.0
	**B3-b**	1.5	38.3	0.98 ± 0.01	0.61 ± 0.01	75.85 ± 0.3	72.1
	**B4-a**	4.5	39.2	1.26 ± 0.01	0.83 ± 0.02	82.6 ± 0.2	80.1
	**B4-b**	6.8	39.2	1.19 ± 0.01	0.72 ± 0.01	82.5 ± 0.3	79.8
	**B6-a**	4.5	38.2	1.16 ± 0.01	0.74 ± 0.03	79.9 ± 0.2	82.6
	**B6-b**	6.8	38.2	1.26 ± 0.02	0.84 ± 0.02	82.2 ± 0.3	83.3
	**B8-B12**	4.5	37.4 ± 0.7	1.14 ± 0.01	0.67 ± 0.01	n/a	n/a
PVDF 2	**B2-f**	4.5	38.6	1.07 ± 0.02	0.66 ± 0.03	77.5 ± 0.2	70.6
	**B2-g**	6.8	38.6	1.16 ± 0.01	0.79 ± 0.01	77.5 ± 0.2	72.6
	**B2-h**	9.0	38.6	1.25 ± 0.01	0.90 ± 0.01	n/a	n/a
	**B5-a**	4.5	38.2	1.14 ± 0.03	0.75 ± 0.01	79.1 ± 0.2	72.8
	**B5-b**	6.8	38.2	1.28 ± 0.03	0.91 ± 0.01	81.2 ± 0.3	75.9
	**B7**	4.5-6.8	38.2	1.16 ± 0.01	0.73 ± 0.02	n/a	n/a

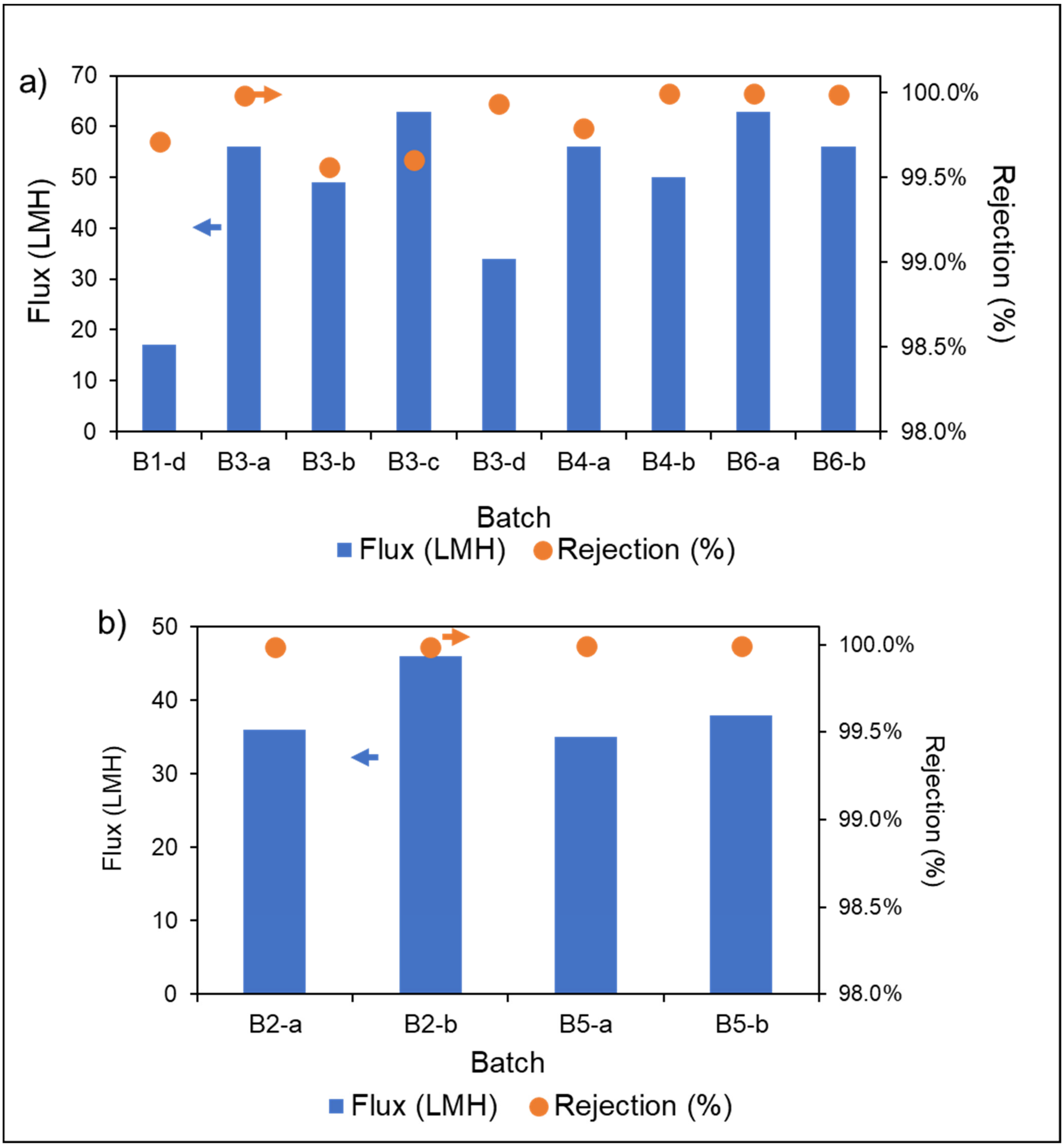

Figure S4. Flux and Rejection of VMD tests in each batch. All tests were performed for a time ≥ 1 hr and using 0.5-in modules. Descending values based on flux, L/m^2.hr (LMH). (a) Fibers made with PVDF 1 dope; (b) Fibers made with PVDF 2 dope.
